# App-Based Myofunctional Therapy to Improve Adherence and Outcomes of Mandibular Advancement Devices in Moderate Obstructive Sleep Apnea: Multicenter Randomized Trial Protocol

**DOI:** 10.2196/80521

**Published:** 2025-12-19

**Authors:** Juan Antonio Ibañez-Rodríguez, Carlos O'Connor-Reina, Ricardo Lucas, Laura Rodriguez-Alcala, Eduardo Correa, Jose Maria Ignacio, Patricia Fernandez-Sanjuan, Peter Baptista, Guillermo Plaza

**Affiliations:** 1 Department of Medical Specialties and Public Health, Universidad Rey Juan Carlos, Madrid, Spain; 2 Department of Otolaryngology, Hospital Quironsalud Marbella, Marbella, Málaga, 29603, Spain, 34 952780540; 3 Department of Otolaryngology, Hospital Quironsalud Campo de Gibraltar, Palmones, Cadiz, Spain; 4 Department of Dentistry, Hospital Quironsalud Marbella, Marbella, Málaga, Spain; 5 Department of Pulmonology, Hospital Quironsalud Marbella, Marbella, Málaga, Spain; 6 Department of Dentistry, Hospital Universitario Sanitas La Zarzuela, Madrid, Spain; 7 Department of Otolaryngology, Al Zahra Hospital, Dubai, United Arab Emirates; 8 Department of Otolaryngology, Hospital Universitario de Fuenlabrada, Madrid, Spain; 9 Otolaryngology Department, Hospital Universitario Sanitas La Zarzuela, Madrid, Spain

**Keywords:** obstructive sleep apnea, myofunctional therapy, mandibular advancement device, adherence, telemedicine, airway gym, digital health, IOPI, sleep quality, Iowa Oral Performance Instrument

## Abstract

**Background:**

Mandibular advancement devices (MADs) are a valid alternative for patients with moderate obstructive sleep apnea (OSA) who do not tolerate continuous positive airway pressure. Despite their efficacy, both adherence and long-term results remain a challenge. Myofunctional therapy (MT) has shown potential to improve upper airway muscle tone and reduce OSA severity, but its role as an adjunct to MAD treatment is still unclear. This protocol evaluates whether app-based MT (Airway Gym) can improve adherence and clinical outcomes in patients with moderate OSA treated with MADs. We describe the study design.

**Objective:**

This study aims to evaluate whether a mobile app–based MT program (Airway Gym) can improve adherence to MAD therapy and related sleep parameters in patients with moderate OSA treated with MADs.

**Methods:**

This multicenter, prospective, randomized, controlled study included adults aged 18‐75 years with moderate OSA (apnea–hypopnea index 15‐30). Participants were randomized into four groups: (1) placebo exercises, (2) MT only, (3) MAD only, and (4) MAD plus MT. Outcomes included changes in the apnea–hypopnea index, oxygen desaturation index, sleep quality (Epworth and Pittsburgh scores), and tongue strength/endurance measured with the Iowa Oral Performance Instrument and a tongue digital spoon. Adherence was monitored via telemedicine and sleep diaries.

**Results:**

Recruitment began in February 2024 across participating centers. Data collection was planned to continue until December 2025, after which the final analyses were performed. The protocol described the study procedures up to the most recent administrative review conducted in January 2025.

**Conclusions:**

This protocol describes the rationale and methodology of a randomized trial evaluating app-based MT as an adjunct to MAD therapy in patients with moderate OSA. Full study results will be reported after the final analysis.

## Introduction

Obstructive sleep apnea (OSA) is a multifactorial and prevalent condition that affects up to 20% of the adult population [1] and is characterized by repeated episodes of upper airway collapse during sleep [2]. This results in oxygen desaturation, fragmented sleep, and increased cardiovascular and neurocognitive risk [3]. Although continuous positive airway pressure (CPAP) remains the first-line treatment, many patients are unable or unwilling to adhere to long-term use, and alternative therapeutic strategies are needed [4].

Mandibular advancement devices (MADs) are a well-established option for patients with mild to moderate OSA [5]. These devices function by advancing the mandible and tongue forward, which thereby increases the patency of the upper airway and reduces its collapsibility [6]. Despite their efficacy, adherence to MAD therapy is inconsistent, and the long-term use rate is 60%‐70%. The long-term adherence depends on each patient’s characteristics and device type [7], and significant morbidity is associated with its use. Studies [8-10] have described potential adverse effects associated with the long-term use of MADs, including gradual dental and skeletal changes such as decreased overjet and overbite, forward displacement of molars, and modifications of intercanine and intermolar relationships [11]. Some patients develop changes in dental inclination, including lingual tilting of the upper incisors and labial tilting of the lower incisors [12]. Skeletal alterations, such as posterior mandibular rotation, increased lower facial height, and vertical changes in the condylar position, have also been reported [10]. Although most studies have not reported significant structural damage to the temporomandibular joint, occasional reports of joint sounds, bite changes such as a posterior open bite, and mild discomfort highlight the need for regular monitoring [13]. In general, these side effects tend to be minor and develop slowly, especially when the appliance is properly adjusted and clinical supervision is maintained.

Myofunctional therapy (MT) is an emerging behavioral treatment based on oropharyngeal exercises aimed at improving the tone and coordination of muscles involved in breathing, swallowing, and speech [14]. Recent studies have shown that MT can significantly reduce the apnea–hypopnea index (AHI) and improve sleep quality in adults with OSA [15,16]. This study is in line with recent national evaluations in Spain that have endorsed the safety and usefulness of MT for treating OSA in selected patients [15]. For the first time, a mobile app—Airway Gym—[16] has been officially recognized by a national health authority as a valid therapeutic tool for OSA. This recognition places Spain at the forefront of integrating digital health into routine care for sleep-related breathing disorders. MT has also been proposed as a supportive intervention for improving tolerance to other treatments such as CPAP [17]. However, its potential role in improving adherence to and therapeutic efficacy of MADs has not been investigated.

The present protocol outlines a multicenter, randomized, prospective controlled study designed to assess whether combining MAD therapy with MT—delivered through the Airway Gym mobile app—can improve both adherence and treatment outcomes in patients with moderate OSA. In addition, the study will evaluate whether baseline oropharyngeal muscle performance, assessed using the Iowa Oral Performance Instrument (IOPI) and a tongue digital spoon (TDS) device, is associated with treatment success after the 3-month intervention period [18]. The primary objective is to assess whether app-based MT enhances adherence to MAD use in patients with moderate OSA. This protocol focuses on the early adaptation phase of MAD therapy, as short-term adherence during the first 3 months has been identified as a critical determinant of long-term treatment continuation and tolerance. Previous studies evaluating early adherence to CPAP and MAD therapy have shown that most barriers to sustained use emerge during this initial period [17].

## Methods

### Study Design

This protocol describes a multicenter, prospective, randomized controlled pilot clinical trial designed to assess the influence of MT on the adherence and efficacy of MAD in patients with moderate OSA. The study was conducted at the Departments of Otolaryngology and Pulmonology of Hospitales Quirónsalud Marbella, Malaga, and Campo de Gibraltar, Cadiz, Spain.

**Figure 1.**
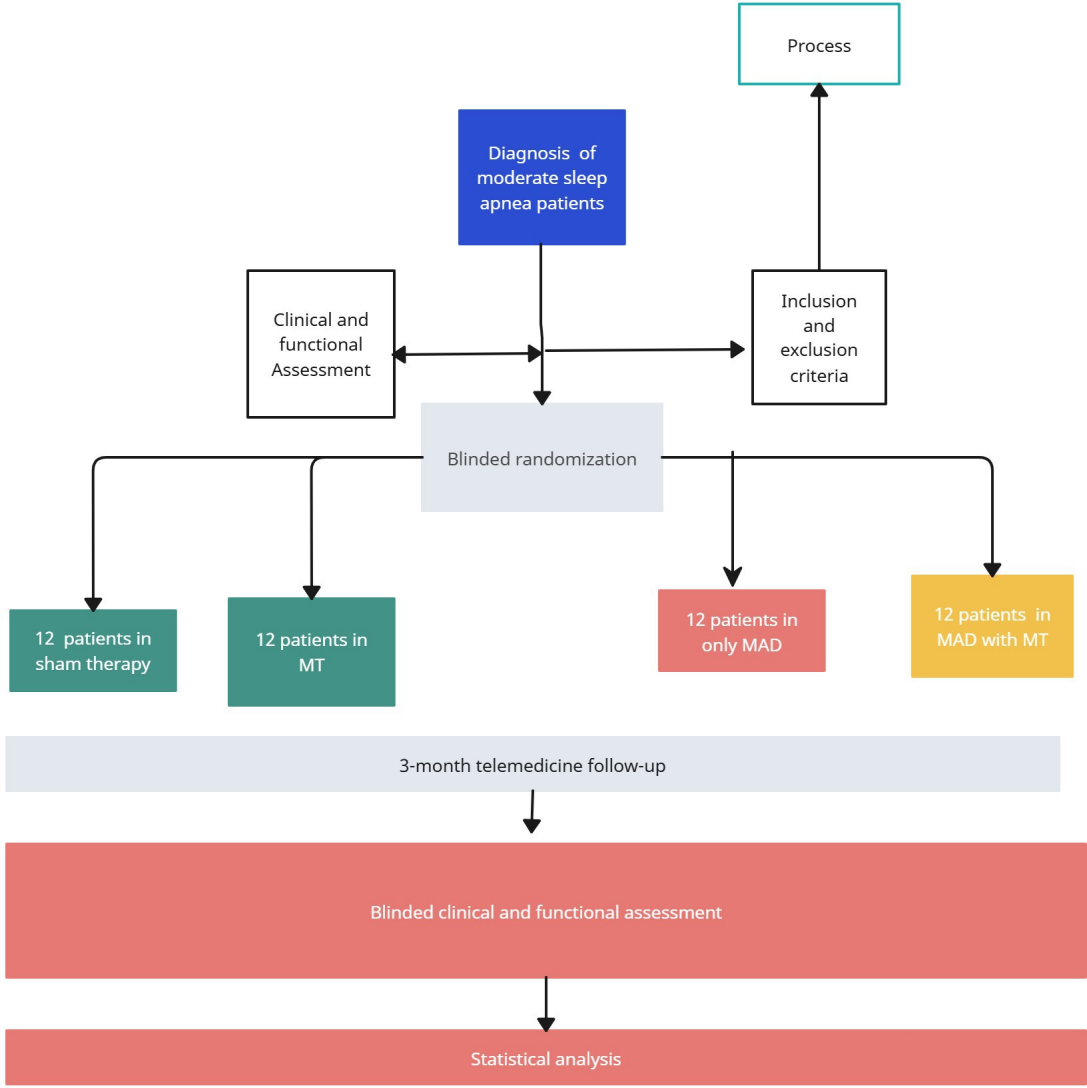
Flowchart of the study design illustrating the random allocation of 48 patients into four parallel groups: (1) placebo orofacial exercises, (2) myofunctional therapy (MT) using the Airway Gym app, (3) mandibular advancement device (MAD) only, and (4) MAD combined with MT. All patients were evaluated at the baseline and after 3 months of intervention using telemedicine monitoring and standardized outcome assessments.

The protocol was approved by the Research Ethics Committee of the Hospital Provincial de Málaga (AWAGAP-2023‐1) and complies with the Declaration of Helsinki and the General Data Protection Regulation (EU) 2016/679. The study is registered with ISRCTN (ISRCTN13128082) [19]. A flowchart summarizing the study design, group allocation, intervention, and follow-up is provided in Figure 1.

Patients aged 18 to 75 years with a confirmed diagnosis of moderate OSA, defined as an AHI between 15 and 30 events per hour, were eligible. All participants required a favorable indication for MAD therapy following drug-induced sleep endoscopy (DISE) [20] and had to provide written informed consent.

### DISE-Based Selection Criteria

All participants underwent DISE as part of the preenrollment assessment to determine the suitability for MAD therapy. The procedure was performed under standardized sedation following the VOTE classification (Velum, Oropharynx, Tongue base, Epiglottis).

Candidates were considered eligible if DISE revealed a predominantly anteroposterior or concentric collapse at the velum or oropharyngeal level that improved with a mandibular advancement (jaw-thrust) maneuver. Subjects with complete concentric collapse at the velum that did not improve with jaw thrust, isolated epiglottic collapse, or predominant lateral wall collapse were excluded. This ensured that all enrolled participants had a favorable DISE pattern predictive of a good response to MAD therapy.

These selection criteria are consistent with previous studies showing that DISE findings, particularly improvement of anteroposterior collapse with a mandibular advancement maneuver, predict a favorable response to oral appliance therapy [21].

Patients were included only if they fulfilled all of the following inclusion criteria and none of the exclusion criteria.

#### Inclusion Criteria

The study included the following inclusion criteria: (1) adults aged 18‐75 years, (2) a diagnosis of moderate OSA (AHI 15‐30) confirmed by polysomnography, (3) no prior treatment for OSA (CPAP, MAD, upper airway surgery, or MT), (4) a favorable anatomical indication for MAD use based on DISE findings, (5) anatomical conditions compatible with the use of an MAD, (6) access to a smartphone and ability to use mobile apps, (7) the willingness and ability to complete daily sleep diary and follow-up assessments, and (8) the provision of signed informed consent.

#### Exclusion Criteria

The study included the following exclusion criteria: (1) the presence of bad nasal patency and the presence of nasal obstruction: patients were excluded if nasal endoscopy revealed bilateral obstruction exceeding approximately 50% of the nasal airway (eg, severe septal deviation, turbinate hypertrophy, or nasal valve collapse) that could interfere with MAD fitting or with the performance of MT. (2) Neurological, neurodegenerative, or cognitive disorders affecting adherence, (3) malformations or edentulism impeding adaptation to the MAD, (4) temporomandibular joint dysfunction, (5) ankyloglossia or lingual restrictions preventing orofacial exercise performance, (6) the inability to follow instructions or participate in telemedicine sessions, (7) active oncological disease, (8) a history of alcohol dependence or other behavioral disorders, (9) uncontrolled psychiatric illness, (10) the lack of access to a reliable internet connection or inability to operate mobile apps, and (11) patients currently participating in a structured weight loss program or those with unintentional weight changes exceeding 5% of body weight within the previous 3 months.

All eligibility criteria were evaluated and confirmed by both an otolaryngologist and a dental sleep specialist before randomization.

### Randomization and Groups

A total of 48 patients were randomized into the following four parallel groups (n=12 each): (1) Group 1: placebo (sham orofacial exercises); (2) Group 2: MT using the Airway Gym app; (3) Group 3: MAD only; and (4) Group 4: combined MAD+MT.

Patients were randomized using a computer-generated block randomization sequence with a 1:1:1:1 allocation ratio to ensure balanced distribution across the four intervention groups. The sequence was stratified by the study center to minimize intersite variability and was generated using an online tool (Sealedenvelope.com). Allocation concealment was achieved using sequentially numbered, opaque, sealed envelopes that had been prepared by an independent researcher not involved in patient enrollment or outcome assessment.

All MADs were custom-fitted (NOA device, OrthoApnea, Málaga, Spain) and adjusted to the maximum comfortable mandibular protrusion.

### Interventions

#### Myofunctional Therapy

**Figure 2.**
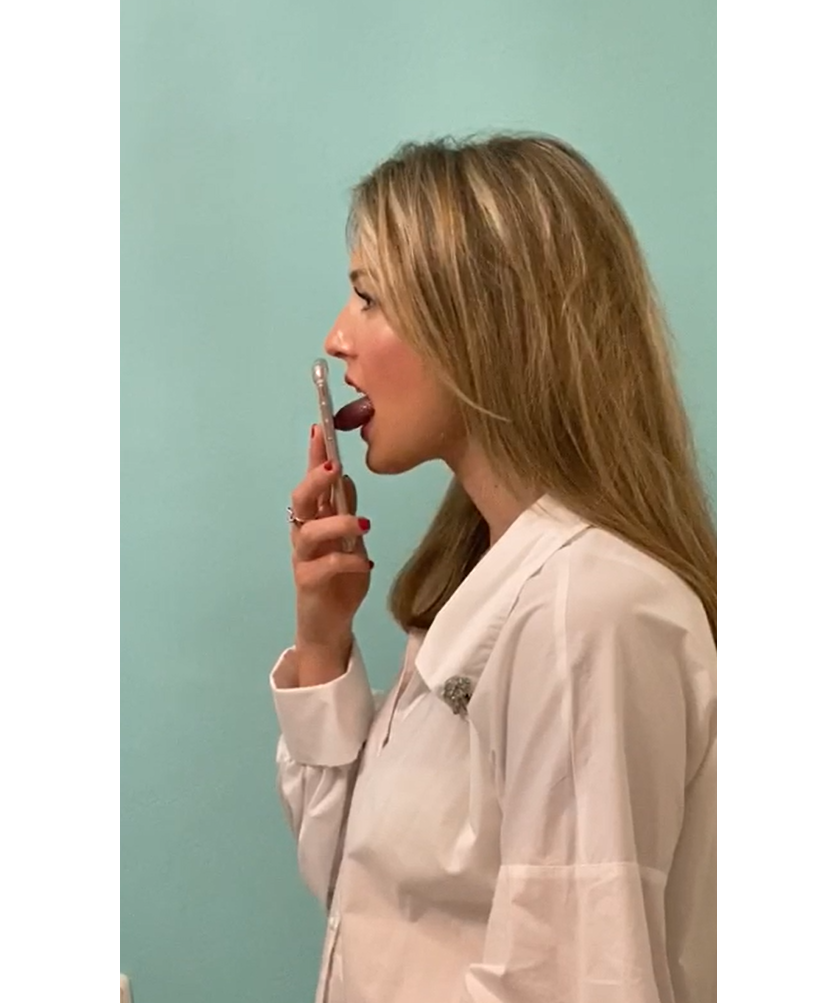
Still image from the app-based myofunctional therapy video (Airway Gym) used during the intervention period. The image shows a patient performing an oropharyngeal exercise as part of the structured and remotely supervised myofunctional therapy protocol delivered via the Airway Gym mobile app.

Participants in the MT groups followed a structured oropharyngeal exercise regimen using the Airway Gym app for 3 months, during which telemonitoring and weekly follow-up were performed [22] (Figure 2).

#### Mandibular Advancement Device

**Figure 3.**
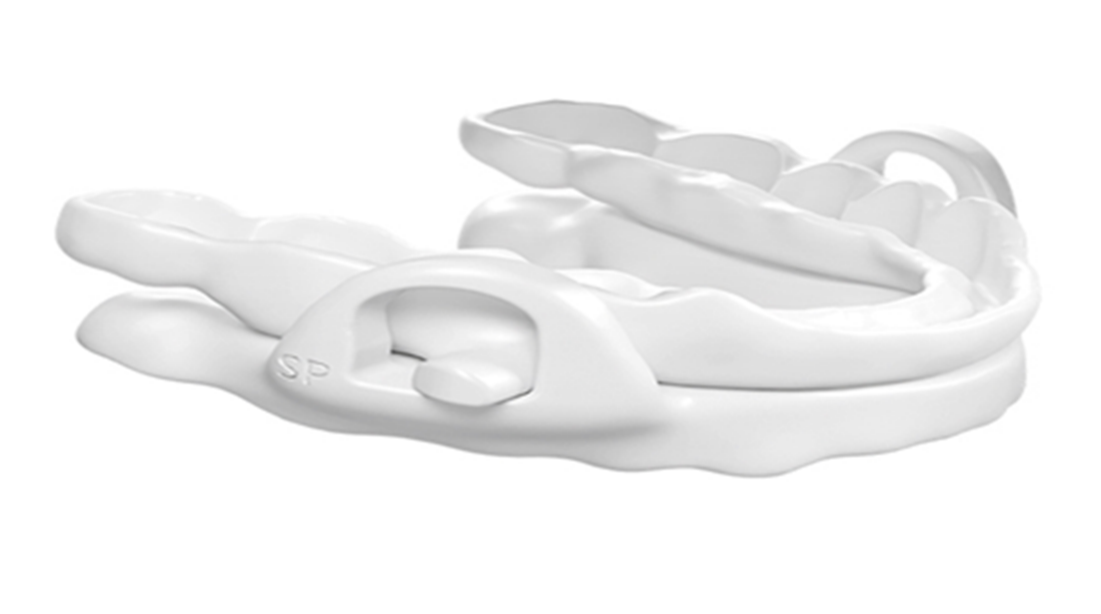
Mandibular advancement device used in the study.

Participants assigned to MAD therapy were fitted with the NOA device (OrthoApnea) [23], a custom-fabricated, two-piece appliance designed to optimize mandibular protrusion and patient comfort. The device allows for gradual titration and lateral movement and is custom manufactured to each patient’s individual craniofacial anatomy. All devices used in the study were generously provided by OrthoApnea for research purposes. Devices were fitted by dental sleep medicine specialists and monitored periodically for side effects and adjustment (Figure 3).

#### Placebo Therapy

The placebo protocol consisted of simple, low-intensity oral and facial movements intentionally designed to avoid activation of the key oropharyngeal and tongue muscles involved in airway function. The exercises included gentle lip gestures, light tongue repositioning without resistance, and relaxed mandibular movements performed without sustained contraction. To ensure neutrality, the placebo sequence was developed in collaboration with experienced myofunctional therapists and ear, nose, and throat specialists. It was pilot-tested in a small group of volunteers to confirm that it produced no change in tongue strength measured by the IOPI and no subjective improvement in orofacial muscle tone. This process ensured that the placebo protocol provided a nontherapeutic control condition for the trial.

### Outcome Measures

#### Primary Outcomes

The primary outcome of the study is adherence to MAD therapy, expressed as the mean hours of use per night and the number of nights per week the device is used.

Adherence was evaluated using structured sleep diaries, telemedicine follow-up, and digital monitoring data (for MT via the Airway Gym app).

This primary endpoint directly reflects the main objective of the study: to determine whether app-based MT improves adherence and acceptance of MAD treatment in patients with moderate OSA.

#### Secondary Outcomes

Secondary outcomes include changes in objective sleep parameters and functional measures, including (1) AHI (events per hour of recording); (2) oxygen desaturation index (ODI; events per hour of recording); (3) minimal nocturnal oxygen saturation (min SpO_₂_); (4) tongue and buccinator strength measured using the IOPI and TDS; (5) sleep quality and daytime sleepiness, assessed using the Pittsburgh Sleep Quality Index, Epworth Sleepiness Scale, and a visual analog scale (VAS) for discomfort; and (6) adverse effects associated with MAD and MT use.

**Figure 4.**
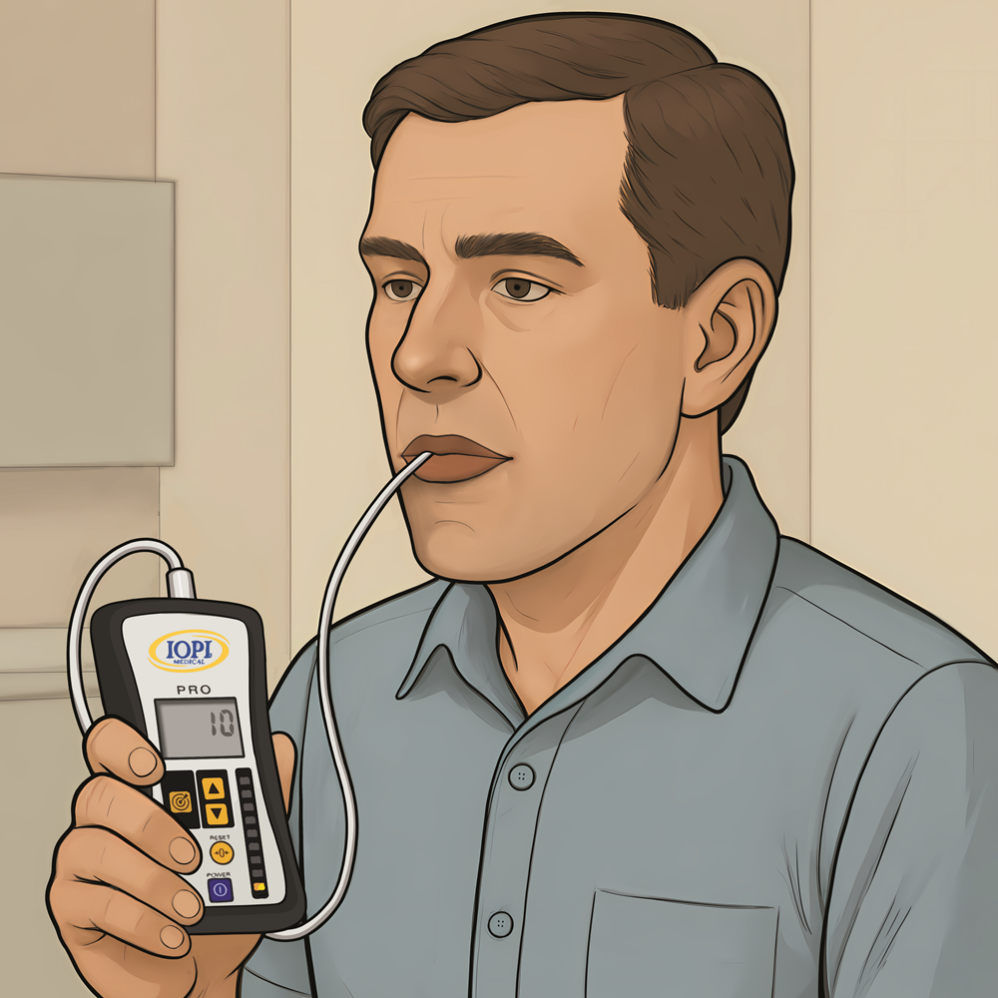
Measurement of tongue strength using the Iowa Oral Performance Instrument (IOPI). The IOPI is used to objectively assess tongue pressure in kPa during isometric compression of an air-filled bulb placed on the tongue.

**Figure 5.**
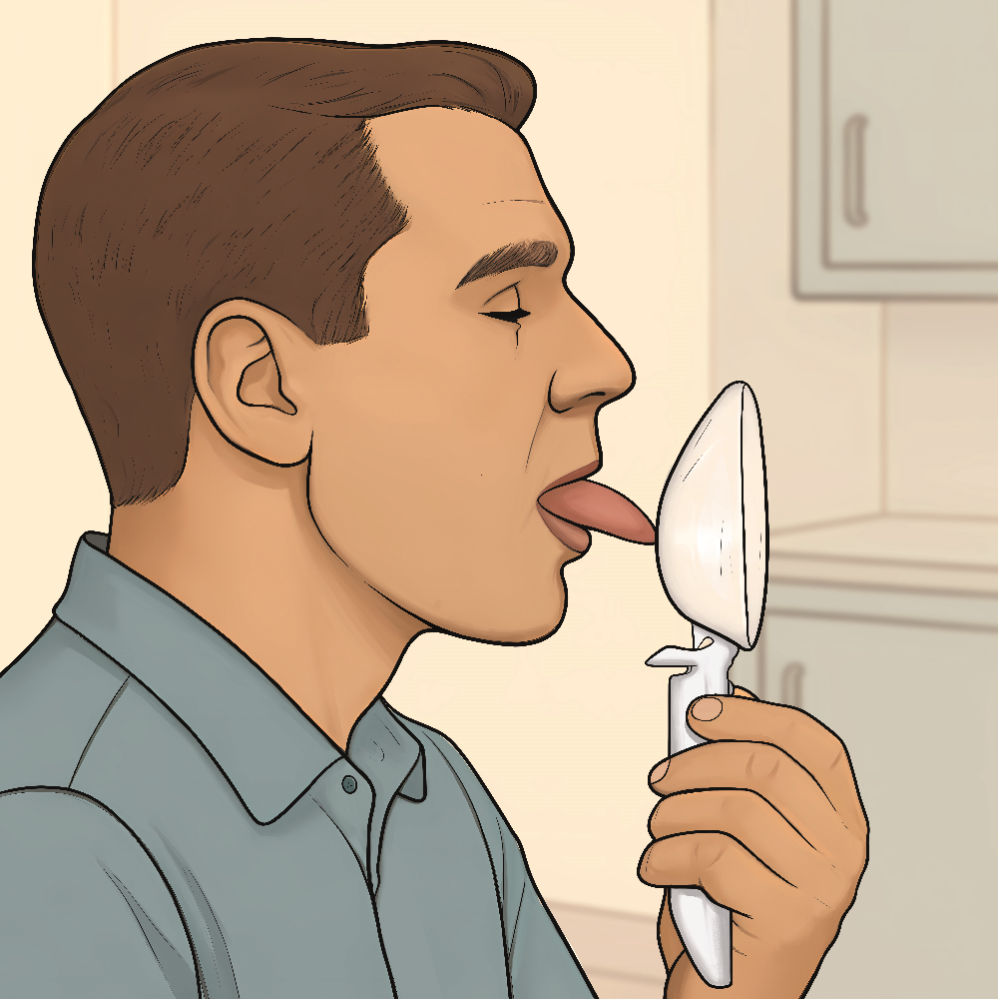
Assessment of anterior tongue pressure using a digital spoon. This tool allows low-cost quantification of anterior lingual force (g/cm²) and can detect changes in oropharyngeal muscle tone over time.

Baseline and 3-month postintervention measurements included anthropometric data (BMI, neck and waist circumference), polygraphy, orofacial myofunctional evaluation with scores [24], IOPI score (kPa) [18] (Figure 4), and TDS (g/cm^2^) [25] (Figure 5).

Polygraphy was performed for each participant using a type III sleep study device (SOMNOtouch, SOMNOmed). During polygraphy, the following parameters were evaluated for this study: mean oxygen saturation and lowest oxygen saturation, time of sleep spent with blood oxygen saturation <90% (SpO_2_<90), and the AHI. The AHI is described as the total number of apnea and hypopnea events per hour of recording in an overnight sleep study. Apnea is defined as a ≥90% decrease in airflow for at least 10 s and hypopnea as a reduction of respiratory signals for ≥10 s, associated with a minimum of 3% of oxygen desaturation [26].

A structured daily sleep diary was used to monitor self-reported side effects, hours of device use, and perceived discomfort, including any mandibular, dental, or temporomandibular joint symptoms. Patients completed a VAS to rate their pain or discomfort and recorded any adverse events associated with the use of the MAD or MT.

### Study Procedures

At the baseline, all participants underwent an otorhinolaryngology examination, DISE, and functional orofacial evaluation. Devices and exercises were introduced at the initial visit. After 3 months, patients underwent a repeat sleep study and functional testing and completed the questionnaires.

Adherence was monitored using a structured sleep diary to record their daily MAD use, exercise compliance, and sleep-related variables (Table 1).

The schedule of enrollment, interventions, and assessments is summarized in Figure 4, following the SPIRIT (Standard Protocol Items: Recommendations for Interventional Trials) guidelines.

**Table 1.** SPIRIT^a^ figure: schedule of enrollment, interventions, and assessments.

Timepoint	Enrollment	Allocation	Post-allocation (Week 0)	Week 1‐11	Week 12 (End of intervention)	Close-out
Enrollment						
Eligibility screening	X					
Informed consent	X					
Baseline assessments (AHI^b^, ODI^c^, min SatO₂^d^, ESS^e^, PSQI^f^, IOPI^g^, and TDS^h^)	X					
Randomization		X				
Interventions						
Sham MT^i^			X	X	X	
MT alone			X	X	X	
MAD^j^ alone			X	X	X	
MAD + MT			X	X	X	
Assessments						
Adherence (telemonitoring and diaries)				X	X	
Sleep study (AHI, ODI, and min SatO₂)			X		X	
IOPI and TDS			X		X	
ESS, PSQI, and VAS^k^			X		X	
Adverse events				X	X	
Close-out						X

a SPIRIT: Standard Protocol Items: Recommendations for Interventional Trials.

b AHI: apnea–hypopnea index.

c ODI: oxygen desaturation index.

d SatO_2_: saturated oxygen.

e ESS: Epworth Sleepiness Scale.

f PSQI: Pittsburgh Sleep Quality Index.

g IOPI: Iowa Oral Performance Instrument.

h TDS: tongue digital spoon.

i MT: myofunctional therapy.

j MAD: mandibular advancement device.

k VAS: visual analog scale.

### Sample Size Calculation

The sample size calculation was primarily based on detecting moderate differences in adherence to MAD therapy (hours per night) between groups, which represents the primary outcome of this study. Secondary analyses explored changes in AHI, ODI, and other physiological measures as supportive efficacy end points. This study was conceived as a multicenter exploratory randomized trial including four parallel treatment groups with assessments at baseline and after 3 months.

These assumptions were informed by previous studies reporting mean reductions in AHI following MT [14] and variability in MAD response and adherence [5]. Considerations regarding the variability of AHI measurement were also based on Pevernagie et al [26].

The power calculation was based on a repeated-measures design (4 groups×2 time points), assuming a two-sided *α*=.05 and within-subject correlations between 0.5 and 0.7. Under these conditions, a total sample of 48 participants provides approximately 80% to 87% power to detect a moderate interaction effect (Cohen *f*=0.30-0.34) for the primary outcome (adherence) and for exploratory analyses of AHI and related parameters. *Power estimation was performed using G* Power 3.1 software [27]. This analysis was powered primarily for adherence, while physiological end points (AHI, ODI, min SpO_₂_) were analyzed as exploratory secondary outcomes.

In practical terms, this sample size offers around 80% power to detect standardized mean differences (g=0.95‐1.10) in change scores between groups. For AHI, this translates to a difference of roughly 8 to 10 events per hour, while for adherence (hours per night of MAD use), it allows the detection of differences of approximately ≥1.5 hours per night assuming a standard deviation of 1.8 hours. For the binary adherence outcome (≥4 hours per night on ≥70% of nights), the study retains ~80% power to identify group differences around 75% versus 35% to 40%.

Although modest in size, this design provides adequate sensitivity to identify meaningful changes and to yield variance estimates that will guide the power calculation of a future large-scale confirmatory trial.

### Statistical Analysis

Descriptive statistics (mean and standard deviation or median and interquartile range) were used to characterize the study population. Between-group differences were analyzed using ANOVA for normally distributed continuous variables and Kruskal–Wallis tests for nonparametric data. For categorical variables, the chi-squared test or Fisher exact test was applied as appropriate.

A prespecified analytic framework was established prior to data analysis following the structure defined in the ISRCTN preregistration. Although a full statistical analysis plan was not separately published, all primary and secondary end points, analytic strategies, and handling of missing data were defined before unblinding.

When the overall group effect is significant, post hoc pairwise comparisons were performed using Bonferroni correction (or Dunn test for nonparametric data) to control for alpha inflation. Paired comparisons (baseline vs 3 mo within each group) were analyzed using the paired *t* test or Wilcoxon signed-rank test.

Correlations between adherence (primary outcome) and physiological parameters (AHI, ODI, min SpO_₂_, IOPI, and TDS) were assessed using Spearman’s rank correlation. All tests were 2-tailed, and statistical significance was set at *P*<.05 after correction for multiple comparisons.

The results were presented with adjusted *P* values and 95% CIs where applicable. Given the exploratory design and modest sample size, the analyses are intended to generate hypotheses for future confirmatory studies rather than establish definitive causal inferences.

Analyses were conducted using Epi Info (CDC) and IBM SPSS Statistics v.28.0 (IBM Corp).

### Participants

Eligible participants were adults aged 18 to 75 years with moderate OSA (AHI 15‐30), confirmed by polysomnography, with favorable anatomical features for MAD use based on DISE. Exclusion criteria included cognitive impairment, temporomandibular dysfunction, severe malocclusion, ankyloglossia, or inability to use mobile apps.

### Randomization and Groups

Forty-eight patients are to be randomized into four groups (n=12 per group): (1) placebo exercises, (2) MT only, (3) MAD only, and (4) MAD+MT. Randomization is performed using computer-generated block sequences and stratified by site.

### Interventions

The MAD used was the NOA device (OrthoApnea), fitted to each patient’s maximum comfortable protrusion. MT was delivered via the Airway Gym mobile app, including structured oropharyngeal exercises for 12 weeks with weekly telemonitoring. The placebo group performed nonfunctional movements. Adherence to MAD and MT was recorded via sleep diaries.

### Outcomes

Primary outcomes included changes in AHI, ODI, and min SpO_₂_. Secondary outcomes were adherence to MAD (hours per night), tongue strength and endurance (measured by IOPI and TDS), and subjective sleep quality (Epworth Sleepiness Scale, Pittsburgh Sleep Quality Index, and VAS for discomfort).

### Adherence Monitoring

Adherence to MT is monitored digitally through the Airway Gym app, which automatically records the number, duration, and completion of each exercise session for every participant. These data provide an objective record of engagement with the therapy. For participants using MADs, adherence is also verified through telemedicine follow-ups and structured clinical interviews.

In addition, participants complete daily sleep diaries to document their perceived use and sleep quality. While sleep diaries may be subject to recall bias and tend to overestimate adherence, their inclusion is consistent with prior studies in behavioral sleep medicine and provides complementary subjective data on patient perception and compliance.

Although sleep diaries are subject to recall bias, adherence to MT is objectively recorded through the Airway Gym app, which automatically logs daily session time, duration, and completion for each participant. Adherence to MAD use is further verified during structured telemedicine visits, providing an additional layer of supervision to minimize overestimation. At the time of study initiation, commercially available MADs with embedded objective adherence sensors were not yet widely accessible in our health care system; therefore, objective device-based adherence monitoring could not be implemented in this exploratory trial.

### Data Collection and Analysis

Assessments were performed at baseline and at 3 months. Polygraphy was used to assess sleep parameters. Orofacial function was evaluated using orofacial myofunctional evaluation with scores protocol (OMES), IOPI (in kPa), and TDS (g/cm²). Descriptive statistics and group comparisons were performed using ANOVA, Kruskal–Wallis, or nonparametric tests as appropriate. A significance level of *P*<.05 was applied.

### Bias Control and Data Management

To prevent performance and detection bias, clinical investigators responsible for participant follow-up, MAD adjustments, and data entry were completely independent from the development of the Airway Gym app. All outcome assessors (polygraphy technicians, IOPI/TDS operators, and questionnaire administrators) were blinded to group allocation. Standardized telemedicine scripts and fixed follow-up schedules were used across all study sites to ensure procedural uniformity regardless of treatment arm.

All outcome data (polysomnographic variables, IOPI measurements, adherence records, and questionnaire scores) are collected and coded by local investigators who are not affiliated with the app’s development. Data are entered into a centralized database managed by an independent statistician blinded to group allocation. Analyses of AHI, ODI, and adherence are performed using deidentified datasets.

Although one investigator is the inventor and scientific advisor of the Airway Gym app, he did not participate in recruitment, data collection, adherence verification, polygraphy interpretation, or statistical analysis. Data were stored in a centralized, access-restricted database managed by the institutional research office, which also performed routine external monitoring of data integrity. Statistical analyses were carried out by an independent biostatistician blinded to group allocation.

### Ethical Considerations

The study was approved by the Ethics Committee of Hospital Provincial de Málaga (approval code: AWGAP-2023-1; date: January 17, 2023) and conducted in accordance with the Declaration of Helsinki. Written informed consent was obtained from all study participants prior to enrollment. Written informed consent for publication of the identifiable image included in Figure 2 was obtained from the individual depicted.

## Results

Participant recruitment started in February 2024. As of January 2025, enrollment and follow-up assessments are progressing according to schedule. Data collection is expected to conclude in December 2025, after which the final analyses will be carried out once all participants have completed the 3-month intervention period.

## Discussion

### Principal Findings

This study primarily aims to determine whether combining app-based MT with MAD therapy improves adherence and tolerance, with secondary analyses exploring changes in AHI and oropharyngeal function. This protocol is expected to contribute new evidence on the potential of combining app-based MT (Airway Gym) with MAD treatment to enhance adherence and functional outcomes in moderate OSA.

We hypothesize that participants receiving both interventions will show greater adherence to MAD use and more pronounced functional improvement than those receiving either treatment alone or placebo exercises.

### Comparison With Prior Work

This hypothesis is supported by previous studies suggesting that oropharyngeal exercises may enhance upper airway function by improving muscle tone and coordination [14,16,18,21,24,28-31]. The trial assessed changes in tongue strength and control through the IOPI and the TDS to determine whether these improvements are more pronounced in participants undergoing MT compared to those treated with MAD alone.

The study explored whether a potential interaction exists between passive mechanical support (via MAD) and active neuromuscular training (via MT), as suggested by prior research [14].

The study also examined whether the combination of MAD and MT may improve patient-reported tolerance and adherence, particularly in individuals with reduced baseline muscle tone or oral fatigue, which are known factors contributing to poor MAD tolerance [32].

### Strengths and Limitations

An important aspect of this study is the use of a mobile app to deliver the MT protocol. This app-based platform is designed to allow remote supervision, daily monitoring, and structured progression of exercises without the need for in-person visits.

This telemedicine approach is expected to offer a scalable, accessible, and cost-effective model for supporting behavioral therapy in OSA, potentially facilitating better adherence to the intervention.

Previous reports have highlighted challenges in implementing MT in routine care—such as time constraints, cost, and low compliance—suggesting that digital tools may help overcome these barriers and promote broader adoption [16,19].

This digital health model may be particularly valuable in health care settings, with limited access to sleep specialists, such as rural or underserved areas, by facilitating the broader implementation of behavioral interventions.

Additionally, the study aims to objectively quantify oropharyngeal muscle changes resulting from MT to assess its potential functional impact.

Increases in IOPI and TDS scores were analyzed to explore whether MT induces neurofunctional adaptations beyond symptomatic improvement [25].

Since discontinuation of MAD therapy in clinical practice is often due to discomfort rather than lack of efficacy, this study assessed whether strategies that promote neuromuscular adaptation, such as telemedicine MT, can enhance device acceptance.

To the best of our knowledge, this is the first randomized controlled trial designed to evaluate the combined use of an MAD and MT in patients with moderate OSA. It is also the first study to deliver MT through a mobile app–based telemedicine platform, allowing for remote supervision and structured progression of exercises.

This study contributed evidence on the value of a treatment model that integrates both anatomical and functional approaches to improve outcomes in patients with moderate OSA [31].

The study has several anticipated limitations. The sample size, while calculated to detect meaningful clinical effects, may still be affected by interindividual variability. Certain outcomes, such as adherence and discomfort, are based on patient-reported data and may be subject to bias. The 3-month follow-up duration was intentionally selected to capture early adherence patterns and short-term functional outcomes, as this period reflects the initial behavioral and neuromuscular adaptation to MAD and MT. Long-term adherence mechanisms were assessed in future longitudinal extensions of this trial once the full cohort completes extended observation.

Another potential limitation of this protocol is the use of self-reported sleep diaries, which are known to overestimate adherence. However, this limitation is common across most studies evaluating behavioral therapy or MT. In our case, adherence is also tracked digitally through the Airway Gym app and verified by telemedicine review, which mitigates the inherent subjectivity of diary-based reporting.

A further limitation is the absence of an embedded objective adherence sensor for the MAD. Although MT adherence is objectively logged through the Airway Gym platform, MAD use still relies partly on patient-reported sleep diaries, which may overestimate usage. This was mitigated through structured telemedicine verification, but future studies should integrate sensor-equipped MADs to provide fully objective adherence monitoring once such technology becomes more accessible.

Another limitation is that, although mechanisms were implemented to ensure independence in data handling and outcome assessment, the involvement of one investigator as the inventor of the Airway Gym app may raise concerns regarding potential performance bias. To mitigate this, data collection and statistical analysis were conducted entirely by independent personnel, and standardized procedures were applied uniformly across study arms.

### Future Directions

If the combined therapy demonstrates greater adherence and functional benefit, this approach could be tested in larger, longer-term studies to assess sustained clinical impact, cost-effectiveness, and potential craniofacial adaptations. Further research also explored integration with wearable sleep-monitoring technologies to enhance remote follow-up.

### Dissemination Plan

The results of this trial were disseminated through peer-reviewed publications and presentations at national and international otolaryngology and sleep medicine conferences. Summaries were also shared with participating centers and made accessible to patients and the public through institutional communication channels and the Airway Gym platform.

### Conclusions

This multicenter trial aims to generate high-quality evidence on the feasibility and effectiveness of integrating app-based MT into standard mandibular advancement treatment for moderate OSA. The results are expected to inform future clinical guidelines and support the development of digital, patient-centered interventions that enhance adherence and treatment outcomes in sleep-disordered breathing.

## Abbreviations

AHI: apnea–hypopnea index
CPAP: continuous positive airway pressure
DISE: drug-induced sleep endoscopy
IOPI: Iowa Oral Performance Instrument
MAD: mandibular advancement device
MT: myofunctional therapy
ODI: oxygen desaturation index
SPIRIT: Standard Protocol Items: Recommendations for Interventional Trials
TDS: tongue digital spoon
VAS: visual analog scale
VOTE: Velum, Oropharynx, Tongue base, Epiglottis
